# Difficult mask ventilation in general surgical population: observation of risk factors and predictors

**DOI:** 10.12688/f1000research.5131.1

**Published:** 2014-08-27

**Authors:** Davide Cattano, Peter V. Killoran, Chunyan Cai, Anastasia D. Katsiampoura, Ruggero M. Corso, Carin A. Hagberg

**Affiliations:** 1 Department of Anesthesiology, University of Texas Medical School at Houston, Houston, 77030, USA; 2 Division of Clinical and Translational Sciences, , Department of Internal Medicine, University of Texas Medical School at Houston, Houston, 77030, USA; 3 Emergency Department, Anesthesia and Intensive Care Section, “GB Morgagni – L Pierantoni” Hospital, Forli, 47121, Italy

## Abstract

**Background: **There are few predictors of difficult mask ventilation and a simple, objective, predictive system to identify patients at risk of difficult mask ventilation does not currently exist. We present a retrospective - subgroup analysis aimed at identifying predictive factors for difficult mask ventilation (DMV) in patients undergoing pre-operative airway assessment before elective surgery at a major teaching hospital.

**Methods**: Data for this retrospective analysis were derived from a database of airway assessments, management plans, and outcomes that were collected prospectively from August 2008 to May 2010 at a Level 1 academic trauma center. Patients were stratified into two groups based on the difficulty of mask ventilation and the cohorts were analyzed using univariate analysis and stepwise selection method.

**Results: **A total of 1399 pre-operative assessments were completed with documentation stating that mask ventilation was attempted. Of those 1399, 124 (8.9%) patients were found to be difficult to mask ventilate. A comparison of patients with and without difficult mask ventilation identified seven risk factors for DMV: age, body mass index (BMI), neck circumference, history of difficult intubation, presence of facial hair, perceived short neck and obstructive sleep apnea. Although seven risk factors were identified, no individual subject had more than four risk factors.

**Conclusion: **The results of this study confirm that in a real world clinical setting, the incidence of DMV is not negligible and suggest the use of a simple bedside predictive score to improve the accuracy of DMV prediction, thereby improving patient safety. Further prospective studies to validate this score would be useful.

## Introduction

One of the primary responsibilities of an anesthesiologist is to maintain adequate oxygenation and ventilation by maintaining a patent upper airway ^1^. Being able to provide ventilation by bag-mask, in face of a difficult or failed tracheal intubation, can make the difference between serious complications and disability.

In the literature, the incidence of difficult mask ventilation (DMV) varies from 0.08% to 15% depending on the definition used ^1– 4^. Despite its importance, there are few predictors of DMV ^2– 4^ and a simple, objective, predictive score to identify patients at risk of DMV at the bedside does not currently exist.

We present a retrospective-subgroup analysis of patients undergoing preoperative airway assessment before elective surgery ^5^ at a major teaching hospital, to identify predictive factors for DMV and evaluate a composite score value, based on a comprehensive airway assessment and recorded outcomes.

## Materials and methods

Data for this retrospective analysis were derived from a database of airway assessments, management plans, and outcomes collected prospectively from August 2008 to May 2010 at a Level 1 academic trauma center (Memorial Hermann Hospital, Texas Medical Center, Houston, TX, USA). The study was sponsored by an educational grant from the Foundation for Anesthesia, Education and Research (FAER), and other educational funds from the Department of Anesthesiology at University of Texas Medical School at Houston. After obtaining IRB approval (HSC-MS-07-0144) all non-obstetric adult patients presenting for elective surgery requiring general anesthesia, were enrolled in this study ^5^.

A total of 91 residents were involved in the data collection process. Residents were randomized into two groups — an experimental group of residents who used a comprehensive airway assessment form in addition to the existing anesthesia record, and a control group, who only used the existing anesthesia record. For the purpose of the present analysis, only the experiment group data were used for a total of 1339 recorded and attempted bag mask ventilations, graded and assigned to a pre-operative airway assessment ^5^.

DMV was defined as difficulty in maintaining a mask seal and obtaining a satisfactory capnography (end-tidal CO _2_ and tidal volume) ^6^. If mask ventilation was attempted, then its easiness was determined and graded based on a severity score: from easy = 0, oral airway used = 1; to difficult, two handed ventilation = 2, or extraglottic device required = 3. However, the use of neuromuscular blocking agent, type, dosage, time of administration, and rescue was not considered in the analysis.

Descriptive statistics mean ± standard deviation for continuous variables and frequency (percentage) for categorical variables was summarized for all pre-operative patient characteristics. Univariate analysis of comparison between patients with or without DMV was performed using the two sample t-test for continuous variables and the Chi-square test or Fisher exact test for categorical variables. In addition, receiver operating characteristic (ROC) curves were used to assess the discrimination ability of predicting DMV using continuous variables and to determine their best thresholds which maximize the sum of sensitivity and specificity. All dichotomized variables with a p-value <0.10 in univariate analysis were entered into a multivariate logistic regression model. A stepwise selection method was used to identify independent predictors of difficult mask ventilation. The adjusted odds ratios and their 95% confidence intervals (CI) were reported for each independent predictor. The area under a ROC curve or c-statistic was calculated to evaluate the resulting model’s predictive value.

A non-weighted risk score was created by assigning one point to each independent predictor. In addition, a weighted score introduced in Kheterparl *et al.* (2009) was derived based on the coefficients of independent predictors from the logistic regression model ^8^. The comparison between non-weighted and weighted risk scores was evaluated through c-statistic. All statistical analyses were conducted using SAS 9.3 (SAS Institute, Cary, NC, USA). A p-value <0.05 was considered significant.

## Results

A total of 1399 pre-operative assessments were completed with documentation that MV was attempted, an ultimate outcome was graded, and the record was linked to a pre-operative airway assessment. Of 1399 patients, 124 (8.9%) were found to be difficult to mask ventilate (2 and 3, Table 1). Once stratified into two groups based on the difficulty of mask ventilation the cohorts were analyzed.

**Table 1.** Summary statistics for MVEase.

MVEase	Frequency (percentage) N=1399
0 = easy	752 (53.8)
1 = Oral airway used	523 (37.4)
2 = Two handed ventilation	118 (8.4)
3 = Extraglottic device required	6 (0.4)

* Mask ventilation was considered easy for MVEase classes 0 and 1 and difficult for MVEase classes 2 and 3. Local practice patterns often include placement of an oral airway for routine bag mask ventilation.

Based on univariate analysis ( Table 2), a total of eight factors were identified with a p-value <0.05: age, gender, BMI, neck circumference, history of difficult intubation, presence of facial hair, perceived short neck and obstructive sleep apnea (OSA, suspected or diagnosed). The thresholds that maximized the sum of sensitivity and specificity were 47 (year) for age, 35 (kg/m ^2^) for BMI, and 40 (cm) for Neck Circumference by analyzing the ROC curve of each continuous risk factor to predict DMV. In addition to these significant factors, an additional variable capturing the absence of dentition (p=0.09) was included in the subsequent analysis. Entering all these nine factors into a multivariate logistic regression model, seven independent risks factors for DMV were identified using stepwise selection: age of 47 year or older, BMI of 35 kg/m ^2^ or greater, and neck circumference of 40 cm or higher, history of difficult intubation, presence of facial hair, perceived short neck, and OSA; p<0.001; ( Table 3). The model’s c-statistic is 0.75 (95% CI: 0.71-0.79), demonstrating a good discriminating capacity. The adjusted odds ratios are also presented in Table 3.

**Table 2.** Preoperative patient characteristics by DMV status.

Variables	DMV		p-value
	False (MVEase=0,1) N=1275	True (MVEase=2,3) N=124	
**Age (year)** ** ≥47**	46±17 614 (48.2)	49±13 80 (64.5)	0.034 0.001
**Male**	628 (49.3)	78 (62.9)	0.004
**BMI (kg/m ^2^)** **≥35**	29.1±7.2 234 (18.4)	33.2±8.0 46 (37.1)	<0.0001 <0.0001
**NeckCirc** ** ≥40**	39.2±4.8 588(46.1)	42.9±4.7 96 (77.4)	<0.0001 <0.0001
**InterIncisors**	4.7±1.0	4.8±0.9	0.204
**Thyromental**	7.9±1.7	7.9±1.7	0.769
**Sternomental**	15.3±2.3	15.3±2.1	0.757
**HxDiffIntub**	7 (0.6)	4 (3.2)	0.012
**NeckMobGrade** **1** **2,3**	1131 (88.7) 144 (11.3)	106 (85.5) 18 (14.5)	0.284
**Mallampati** **I, II** **III, IV**	1081 (84.8) 194 (15.2)	100 (80.7) 24 (19.4)	0.225
**CSpineAbn**	40 (3.1)	7 (5.7)	0.183
**NoTeeth**	107 (8.4)	16 (12.9)	0.090
**FacHair**	126 (9.9)	29 (23.4)	<0.0001
**FacTrauma**	18 (1.4)	0 (0)	NR
**FullStomach**	6 (0.5)	1 (0.8)	0.479
**NasalDef**	5 (0.4)	1 (0.8)	0.428
**NeckTrauma**	17 (1.3)	3 (2.4)	0.413
**ShortNeck**	69 (5.4)	22 (17.7)	<0.0001
**ObsSA**	198 (15.5)	41 (33.1)	<0.0001
**ResYear** **CA-1, CA-1-2** **CA-2, CA-2-3, CA-3**	980 (76.9) 295 (23.1)	92 (74.2) 32 (25.8)	0.503

NR: not reported due to zero cells. Values are reported as mean±SD and frequency (percentage).

**Table 3.** Seven independent predictors of difficult mask ventilation.

Predictor	β Coefficient	Standard Error	p-value	Adjusted odds ratio (95% Confidence Interval)
Age≥47	0.677	0.205	0.001	1.97 (1.32, 2.94)
BMI≥35	0.737	0.222	0.001	2.09 (1.35, 3.23)
NeckCirc≥40	0.931	0.239	<0.001	2.54 (1.59, 4.05)
HxDiffIntub	1.536	0.692	0.026	4.65 (1.20, 18.02)
FacHair	0.849	0.251	<0.001	2.34 (1.43, 3.83)
Short Neck	0.631	0.291	0.030	1.88 (1.06, 3.32)
ObsSA	0.503	0.223	0.023	1.65 (1.07, 2.56)

**Figure 1.**
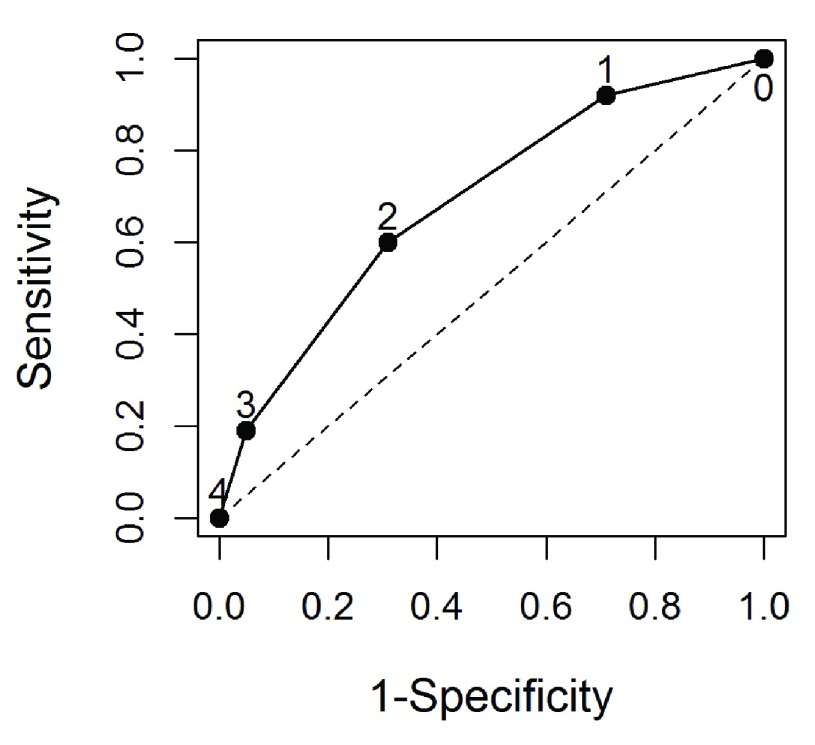
A receiver-operating-characteristic (ROC) curve evaluating the sensitivity and specificity of pre-operative independent risk factors for difficult mask ventilation (DMV). Seven independent predictors for difficult mask ventilation were identified using logistic regression: age of 47 yr or older, BMI of 35 kg/m ^2^ or greater, NeckCirc of 40 or greater, HxDiffIntub, FacHair, short neck and OSA. A risk score for DMV was calculated based on the number of these seven risk factors a patient possessed. The area under the curve was 0.70±0.02.

The seven independent risk factors identified were then applied to all cases where DMV was encountered to evaluate a predictive model for DMV. Although seven risks factors were identified, no individual subject had more than four risk factors. As indicated, non-weighted and weighted risk score were created based on these seven risk factors. The model’s c-statistic based on unweighted score is 0.70 (95% CI: 0.66-0.74) ( Figure 1). Weighted score did not improve the prediction performance, which model’s c-statistic is 0.70 (95% CI: 0.66-0.75). Therefore, we adopted the simple approach of unweighted risk score for the following analysis. The sensitivity, specificity, likelihood ratios, and predictive values were progressively calculated for patients with different number of risk factors ( Table 4). The best cut-off for the number of risk factors was 2, which maximizes Youden’s index ^16^ with sensitivity of 0.65 and specificity of 0.67. Table 5 also shows the distribution frequencies of different number of risk factors and the odds ratio for patients with one, two, or three risk factors relative to a patient with zero risk factors. When compared with zero risk factors, patients with two or more risk factors have an odds ratio of 7.6 (95% CI: 3.4-16.9).

**Table 4.** Diagnostic value of the cut-off for number of risk factors in predicting a difficult mask ventilation.

Cut-off for number of risk factors	Sensitivity	Specificity	Likelihood ratio positive	Likelihood ratio negative	Positive predictive value	Negative predictive value
1	0.94	0.26	1.27	0.23	0.11	0.98
2	0.65	0.67	1.97	0.52	0.16	0.95
3	0.19	0.95	3.80	0.85	0.26	0.92
4	0.00	1.00	N/A	1.0	0.00	0.91

Likelihood ratio positive=Sensitivity/(1-Specificity) Likelihood ratio negative=(1-Sensitivity)/Specificity N/A: not applicable

**Table 5.** Odds ratio of patients with a given risk level (i.e., number of risk factors at 1, 2, 3) to a patient with 0 risk factor.

Number of risk factors	Total patients	Patients with DMV n (%)	Odds Ratio (95% Confidence Interval)
0	337	7 (2.1)	Referrence
1	559	36 (6.4)	3.25 (1.43, 7.38)
2	410	57 (13.9)	7.61 (3.42, 16.93)
3	93	24 (25.8)	16.40 (6.79, 39.57)

## Discussion

For more than three decades, poor airway management was recognized as a serious patient safety concern, emphasizing the need for a careful airway assessment and identifying the predictors for a difficult airway ^6^. Moreover, the airway risk assessment tools in widespread use were mostly focused on one specific aspect of a difficult airway (i.e. difficult laryngoscopy, difficult intubation). In more recent years, this paradigm has shifted to a more functional approach with greater emphasis placed on the overall importance of the airway patency. Indeed, due to early data demonstrating the significant risk of respiratory depression associated with sedation, The Joint Commission and Centers for Medicare and Medicaid Services has implemented policies to ensure evaluation of the risk for a difficult airway prior to procedures. Moreover, the 2013 American Society of Anesthesiology (ASA) Practice Guidelines for Management of the Difficult Airway caution about the risks of a difficult bag-mask ventilation due to upper airway obstruction and recommend an airway risk assessment before every anesthesia procedure is performed ^7^. In this study, we determine that: (1) the reported incidence of DMV was 9%; (2) the reported incidence of DMV in patients with a history of OSA was 17%; (3) seven independent risk factors were identified (age ≥ 47 yr, BMI ≥ 35 kg/m ^2^, neck circumference ≥ 40 cm, history of difficult intubation, presence of facial hair, perceived short neck, history of OSA); (4) the absence of three of these factors allows to reasonably exclude a DMV (likelihood ratio negative: 0.85).

Recent investigations have demonstrated that the incidence and risk factors for DMV are distinct from difficult laryngoscopy (DL) predictors (incidence i.e. ranges from 1.4% ^8^ to 16% ^9^). There are many reasons that can explain these findings: (1) absence of a universally accepted definition of DMV (different definitions lead to different data); (2) obesity and OSA are undoubtedly predictors of DMV, therefore a study done on a population with a high prevalence of obesity will show a higher incidence of DMV from a population with a lower prevalence of obesity; (3) the design of face masks and the technique used are not usually reported, but recent studies highlight their importance for performance and accordingly the reported incidence of DMV ^10, 11^; (4) the influence of neuromuscular block on mask ventilation has been demonstrated, but often these data are missing ^12^.

We confirmed many factors such as age, short neck, facial hair, BMI, but most importantly neck circumference, that have been associated with difficult airway in the obese ^13, 14^ as well as a history of OSA. Interestingly, neck circumference and BMI are also important determinants for OSA screening, which may results in some overlap between OSA and DMV. Our study confirmed that OSA patients are at risk for DMV, calling for a systematic screening for OSA with the aim to identify a category of patients at risk of not only difficult airway, but also of post-operative complications ^15^.

We attempted, indeed, to define a bedside score to predict DMV: our score has the advantage of including objective variables, such as neck circumference, but has a high false positive rate, possibly limiting the usefulness for a large-scale clinical implementation of the score. However with a sensitivity of 92% (using one risk factor, while it drops at lower values for two or more combined risk factors), this score could actually be useful as screening tool, since avoiding the underestimation of unpredicted DMV is far more important than a false positive (particularly in airway management where there are not significant costs attributed to overestimation).

Our study also has other limitations: first, only DMV outcomes were analyzed without consideration for difficult laryngoscopy; second, a large number of records were selectively removed from our analysis because the outcomes were not known, reducing our statistical power and introducing the possibility of selection bias.

The results of this study confirm that in a real world clinical setting, the incidence of DMV is not negligible and suggest the use of a simple bedside predictive score to improve the accuracy of DMV prediction, thereby improving patient safety. Further prospective studies to validate this score would be useful.

## Data availability

Data have been obtained from databases at the Memorial Hermann Hospital, Texas Medical Center, Houston, IRB approval HSC-MS-07-0144. The author can support applications to the Institutional Board to make the data accessible upon individual request.
