# Supplementary material for: Difficult mask ventilation in general surgical population: observation of risk factors and predictors
Source: F1000Res. 2014 Aug 27;3:204. [Version 1] doi: 10.12688/f1000research.5131.1 (PMC4244761; doi:10.12688/f1000research.5131.1)
Supplement: Peer review history

# Peer review: Referee response for version 1

**DOI:** 10.5256/f1000research.5471.r6308

**Published:** 2014-10-17

**Authors:** Adrian Matioc

**Affiliations:** 1 University of Wisconsin School of Medicine and Public Health, William S. Middleton Memorial Veterans Medical Center, Madison, USA

There is today an increasing interest in studying difficult face mask ventilation. Cattano *et al*. present a retrospective study derived from a database built of elective cases. There are several points I want to make:

First, the study used a “real world clinical setting” confirming that in the daily practice difficult face mask ventilation is not a rare occurrence.

Second, it introduced an objective assessment tool for the difficult face mask ventilation: capnography (further comment from the authors regarding this tool would be appreciated).

Third: six out of the seven independent risk factors for the difficult face mask ventilation (age, BMI, neck circumference, presence of facial hair, perceived short neck, OSA) that were identified and then applied to evaluate a predictive model can be easily assessed or inferred with a clinical exam on an unconscious patient thus having probably a role in the outside of the operating room setting too.

Fourth: the study confirms the importance of neck circumference, BMI and OSA in the difficult face mask ventilation paradigm. It is known that these clinical settings are plagued by inspiratory and expiratory obstruction at the soft palate level that does not respond to airway manoeuvres. The soft palate obstruction (relevant for nasal ventilation) can be by passed by committing to oral ventilation (by opening the mouth with an oropharyngeal airway). ^1^ ^2^ It may be appropriate to recommend in these clinical settings a first (optimized) face mask ventilation attempt with an oropharyngeal airway *in situ*.

I have read this submission. I believe that I have an appropriate level of expertise to confirm that it is of an acceptable scientific standard.

## References

[1] SatoYIkedaAIshikawaTIsonoS: How can we improve mask ventilation in patients with obstructive sleep apnea during anesthesia induction?. *J Anesth.*.2013;27(1) : 204152-15610.1007/s00540-012-1520-523212587

[2] BuffingtonCWWellsCMQSooseRJ: Expiratory Upper Airway Obstruction Caused by the Soft Palate during Bag-Mask Ventilation. *Open Journal of Anesthesiology*.2012;2(2) : 20438-4310.4236/ojanes.2012.22010Reference source

# Response

**Published:** 2014-10-17

**Authors:** Davide Cattano

**Affiliations:** Department of Anesthesiology, The University of Texas Medical School at Houston, USA

We are grateful for Dr Matioc comments which endorse our findings while offring an opportunity for further discussion. We do agree with Dr Matioc about the importance of properly monitoring ventilation, which may not always be possible simply by utilizing a capnometry tool, and current investigations are actually looking at other type of devices, particularly in the postoperative arena, where airway obstruction may still occur. In the operating room the presence of capnometry combined with spirometry is a complete tool. Regarding airway adjuncts, I agree that an oral airway cannula should be promptly used, as in fact was one of the limitations of our investigation, since it is part of common airway management pathways, however I would consider also a nasal airway, if available and not concerning trauma, for a better and proper management of ventilation.

# Peer review: Referee response for version 1

**DOI:** 10.5256/f1000research.5471.r5963

**Published:** 2014-08-28

**Authors:** D John Doyle

**Affiliations:** 1 Department of General Anesthesiology, Cleveland Clinic Main Campus, Cleveland, USA

This is an important contribution to the difficult airway literature and will be helpful to clinicians wanting a means to predict that mask ventilation may be a problem following the induction of general anesthesia. While the limitations of the study are correctly stated (see p. 5)  I would add some wording to this section to re-emphasize that this was a retrospective study and that the use of neuromuscular blocking drugs was not part of the analysis. In addition, since a great many cases of difficult mask ventilation vanish following the administration of neuromuscular blocking drugs, I recommend that the discussion on this particular issue should be discussed in a bit more detail, perhaps making reference to one or more of the following recent publications.

Xue FS, Cheng Y, Li RP: Facemask ventilation and neuromuscular blockade in anesthetized patients. Anesthesiology. 2013; **118**(4):991-2. doi: 10.1097/ALN.0b013e3182874628

Richardson MG, Litman RS: Ventilation before paralysis: crossing the Rubicon, slowly. *Anesthesiology.* 2012; **117**(3):456-8. doi: 10.1097/ALN.0b013e318266868f

Engelhardt T, Weiss M: Difficult mask ventilation and muscle paralysis. *Anesthesiology*. 2013; **118**(4):994. doi: 10.1097/ALN.0b013e3182874659.

Priebe HJ: Ventilation before paralysis. *Anesthesiology*. 2013; **118**(4):992-3. doi: 10.1097/ALN.0b013e3182874642.

I have read this submission. I believe that I have an appropriate level of expertise to confirm that it is of an acceptable scientific standard.

# Response

**Published:** 2014-08-28

**Authors:** Davide Cattano

**Affiliations:** Department of Anesthesiology, The University of Texas Medical School at Houston, USA

I am grateful to Dr Doyle for his review, comments and, in particular, for the detailed references.

Indeed the avoidance of muscle relaxation from the analysis was a major "missed" undertaking, since you are completely right about the important effects of muscle relaxation. We made sure that such information was immediately available to the reader as a methodological point, and we kept it short in the discussion to focus on positive findings. It is always of importance to report both positive and negative findings; however, the lack of such related data was missing information rather than negative. We appreciate, however, your comment giving an opportunity for the reader to understand the importance of such a factor. We also need to remind the reader of the interesting aspect that muscle relaxation plays in difficult airway management and how it also plays a role in the DAW algorithms. It is not trivial to consider that our practice includes the establishment of an airway after a mask ventilation is achieved without the utilization of muscle relaxant at first (which we included as standard of care in our investigation) while muscle relaxant is utilized as a rescue for such events.

It is important to consider the evidence value of certain practices though - whether or not they not only make sense, but are of value for a safe and practical airway management. In fact, the controversy - as you elegantly point at with the references provided - is about giving muscle relaxant up front (which was part of anesthesia practice a few years ago). Based on experience, the question is rather: can I use muscle relaxant or not, and should I do an awake intubation (or other airway)? Also the major change that occurred from the recognition of airway related morbidity and mortality in the 80's and 90's and now, is the availability of extraglottic devices and new airway tools that have in fact improved our practice.
